# Association of glial and neuronal degeneration markers with Alzheimer’s disease cerebrospinal fluid profile and cognitive functions

**DOI:** 10.1186/s13195-020-00657-8

**Published:** 2020-08-04

**Authors:** Unnur D. Teitsdottir, Maria K. Jonsdottir, Sigrun H. Lund, Taher Darreh-Shori, Jon Snaedal, Petur H. Petersen

**Affiliations:** 1 Faculty of Medicine, Department of Anatomy, Biomedical Center, University of Iceland, Reykjavik, Iceland; 2 Department of Psychology, Reykjavik University, Reykjavik, Iceland; 3 Department of Psychiatry, Landspitali – National University Hospital, Reykjavik, Iceland; 4 deCODE genetics/Amgen, Inc., Reykjavik, Iceland; 5 Division of Clinical Geriatrics, Center for Alzheimer Research, NVS Department, Karolinska Institutet, Huddinge, Sweden; 6 Memory clinic, Department of Geriatric Medicine, Landspitali - National University Hospital, Reykjavik, Iceland

**Keywords:** Alzheimer’s disease, Cerebrospinal fluid, Neurofilament light, YKL-40, S100 calcium-binding protein B, Glial fibrillary acidic protein, AD biomarker profile, Cognitive domains

## Abstract

**Background:**

Neuroinflammation has gained increasing attention as a potential contributing factor in the onset and progression of Alzheimer’s disease (AD). The objective of this study was to examine the association of selected cerebrospinal fluid (CSF) inflammatory and neuronal degeneration markers with signature CSF AD profile and cognitive functions among subjects at the symptomatic pre- and early dementia stages.

**Methods:**

In this cross-sectional study, 52 subjects were selected from an Icelandic memory clinic cohort. Subjects were classified as having AD (*n* = 28, age = 70, 39% female, Mini-Mental State Examination [MMSE] = 27) or non-AD (*n* = 24, age = 67, 33% female, MMSE = 28) profile based on the ratio between CSF total-tau (T-tau) and amyloid-β_1–42_ (Aβ_42_) values (cut-off point chosen as 0.52). Novel CSF biomarkers included neurofilament light (NFL), YKL-40, S100 calcium-binding protein B (S100B) and glial fibrillary acidic protein (GFAP), measured with enzyme-linked immunosorbent assays (ELISAs). Subjects underwent neuropsychological assessment for evaluation of different cognitive domains, including verbal episodic memory, non-verbal episodic memory, language, processing speed, and executive functions.

**Results:**

Accuracy coefficient for distinguishing between the two CSF profiles was calculated for each CSF marker and test. Novel CSF markers performed poorly (area under curve [AUC] coefficients ranging from 0.61 to 0.64) compared to tests reflecting verbal episodic memory, which all performed fair (AUC > 70). LASSO regression with a stability approach was applied for the selection of CSF markers and demographic variables predicting performance on each cognitive domain, both among all subjects and only those with a CSF AD profile. Relationships between CSF markers and cognitive domains, where the CSF marker reached stability selection criteria of > 75%, were visualized with scatter plots. Before calculations of corresponding Pearson’s correlations coefficients, composite scores for cognitive domains were adjusted for age and education. GFAP correlated with executive functions (*r* = − 0.37, *p* = 0.01) overall, while GFAP correlated with processing speed (*r* = − 0.68, *p* < 0.001) and NFL with verbal episodic memory (*r* = − 0.43, *p* = 0.02) among subjects with a CSF AD profile.

**Conclusions:**

The novel CSF markers NFL and GFAP show potential as markers for cognitive decline among individuals with core AD pathology at the symptomatic pre- and early stages of dementia.

## Introduction

In recent years, a paradigm shift in the research criteria of Alzheimer’s disease (AD) has occurred as the primary focus has shifted from clinical to biological criteria. The emphasis is now on the pathology [1], which is believed to start decades before the appearance of clinical symptoms [2]. The core cerebrospinal fluid (CSF) biomarkers reflecting the hallmarks of AD pathology, extracellular amyloid plaques (Aβ), and neurodegeneration (total tau [T-tau] and phosphorylated tau [P-tau]) have been at the center of this shift and have been extensively studied [3]. Although the diagnostic accuracies of these markers are generally satisfactory [4], their levels are relatively constant in the symptomatic stages of the disease and do not correlate well with the progression of cognitive decline [5–7]. This necessitates the need for exploration of novel biomarkers that help in better understanding the different aspects of AD pathology, its progression, and clinical manifestation.

Increasing evidence shows that inflammation is a contributing factor in the pathogenesis and development of AD and other neurodegenerative diseases [8, 9]. A number of studies show that Aβ toxicity and plaques induce an immune response, including activation of astrocytes and microglia, the immune cells of the brain [10–12]. Furthermore, activation of these cells is also thought to play a role in the formation and progression of neurofibrillary tangles (NFTs), contributing to neuronal dysfunction and loss [13]. Glial activation markers are, therefore, of high interest when it comes to exploring new biomarkers for the diagnosis of dementia.

The glial proteins YKL-40 (also known as chitinase-3-like-1 protein), S100 calcium-binding protein B (S100B), and glial fibrillary acidic protein (GFAP) have previously been associated with AD pathology [14]. All are expressed in astrocytes within the central nervous system (CNS), primarily (YKL-40 and S100B) [15, 16] or exclusively (GFAP) [17]. YKL-40, a chitin-binding glycoprotein and a glial activation marker [18], has been identified inside reactive astrocytes in close proximity to amyloid plaques [19]. YKL-40 expression also correlates with tau pathology in AD brain tissues, demonstrating an association between glial activation and neurodegeneration [20]. S100B is a calcium-binding protein, exerting both intracellular and extracellular functions and has been found to be upregulated in AD tissues [21, 22]. GFAP is a key intermediate filament protein and marker of reactive astrocytes, whose expression has been associated with amyloid plaque load and, to a lesser extent, the number of NFTs [23–25].

Inflammation in the brain and its role in AD can be studied indirectly through the analysis of CSF proteins. Increased levels of CSF YKL-40, S100B, and GFAP have been observed in AD patients compared to healthy controls, although results have not been consistent [26]. The relationship between inflammatory and core AD markers (Aβ, tau) in CSF has also been explored. Previous studies have found a strong positive association between CSF YKL-40 and tau proteins but not between YKL-40 and Aβ_42_ [19, 27–29]. YKL-40 has also been shown to strongly correlate with neuronal degeneration marker neurofilament light (NFL) in CSF [30], further supporting the association between glial activation and neurodegeneration. NFL is mainly located in myelinated axons. Therefore, its levels also reflect white matter changes, with recent studies indicating a potential for this protein as both a diagnostic and progression marker in AD and other neurodegenerative diseases [26, 31]. Few studies have examined the relationship between S100B and GFAP with core AD markers in CSF. Hov et al. [32] found an association between S100B and P-tau but not Aβ_42_ among elective surgery patients free from dementia and delirium. Ishiki et al. [33] did not find an association between CSF GFAP and core markers within a dementia cohort.

Loss of memory is typically among the first clinical symptoms of AD, marking the beginning of cognitive decline. The medial temporal lobe is an early site of tau accumulation, and its dysfunction may underlie episodic memory decline [34]. Other cognitive domains are also involved in AD, such as language, non-verbal episodic memory, and executive functions [35].

In the most recent research criteria from the International Working Group for the diagnosis of AD published in 2014 [36], the diagnosis of prodromal AD requires both the presence of cognitive symptoms and AD signature biomarker profile (increased amyloid positron emission tomography [PET] deposition or the combination of lowered CSF amyloid-β_1–42_ and elevated CSF tau). It is essential for the evaluation of novel biomarkers to examine their relationship with both entities separately, independent of diagnosis. That type of approach could enhance both understanding of the underlying pathology of AD and the sequence of events leading to cognitive impairment. The first aim of this study was to assess the ability of glial (YKL-40, S100B, GFAP) and neurodegeneration (NFL) markers in CSF to discriminate between different CSF profiles (AD and non-AD) among subjects at the symptomatic pre- and early stages of dementia. In addition, the results were compared to the discrimination ability of neuropsychological tests, which are commonly used to aid AD diagnosis. The second aim was to investigate the relationship between the CSF markers with neuropsychological tests reflecting different cognitive domains.

## Methods

### Subjects

**Fig. 1:**
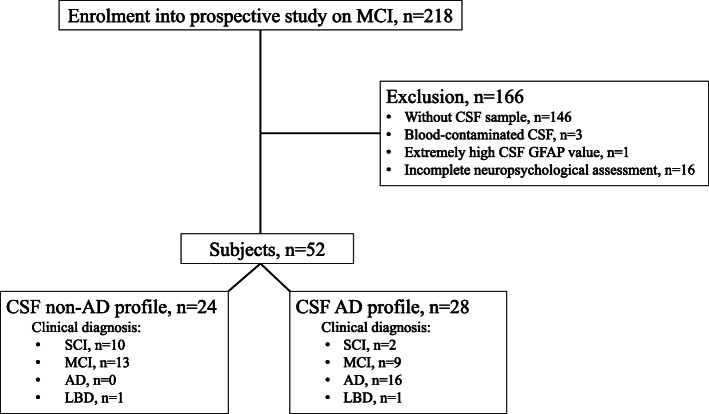
Flow diagram of sample selection

Individuals, referred to The National University Hospital of Iceland Memory Clinic during a 4-year period which had (1) a score between 24 and 30 on the Mini-Mental State Examination (MMSE) and (2) a score of 4.0 or less on the Informant Questionnaire on Cognitive Decline in the Elderly (IQCODE) [37], were invited to join a prospective study on mild cognitive impairment (MCI, *n* = 218). The exclusion criteria were (1) cognitive impairment that, without a doubt, could be explained by a condition other than dementia; (2) difficulties participating due to health or social issues; and (3) residency outside the Reykjavík Capital Area. In entering the study, each subject underwent various assessments, including a standard clinical and neuropsychological assessment and brain magnetic resonance imaging (MRI) for evaluation of medial temporal lobe atrophy (MTA). Lumbar puncture for collection of CSF, which was optional by the requirement of the National Bioethics Committee, was also carried out. For this particular study (Fig. 1), only subjects with CSF samples and complete neuropsychological assessment were selected from the cohort (*n* = 56). The final sample included 52 subjects as four were removed due to excessively high value on CSF GFAP (*n* = 1) or blood-contamination in the CSF sample (*n* = 3). Clinical diagnosis of AD was based on the criteria for probable AD dementia defined by the National Institute on Aging-Alzheimer’s Association (NIA-AA) [38], with evidence of AD pathophysiological processes (based on MTA score or/and analysis of core CSF markers). Patients with Lewy body dementia (LBD) were diagnosed based on the consensus criteria of McKeith et al. [39]. MCI diagnosis required the fulfillment of the Winblad criteria [40], with those not fulfilling the criteria diagnosed as having subjective mild cognitive impairment (SCI).

### CSF collection and analysis

CSF was collected via lumbar puncture with a 22-gauge spinal needle at the L3/4 or L4/5 interspace. Uncentrifuged samples were frozen in 2-ml polypropylene tubes and stored at − 80 °C. Commercially available sandwich enzyme-linked immunosorbent assays (ELISAs) were used for measurements of all proteins. Analyses of core AD markers T-tau (IBL International, Hamburg, Germany) and Aβ_42_ (IBL International, Hamburg, Germany) were carried out in the ISO 15189 accredited medical laboratory MVZ Labor P.D. Dr. Volkmann und Kollegen GbR (Karlsruhe, Germany). Assays for novel markers NFL (Uman Diagnostics, Umeå, Sweden), YKL-40 (Quantikine ELISA Human Chitinase-3–like 1; R&D systems, M.N., USA), S100B (BioVendor GmbH, Heidelberg, Germany), and GFAP (BioVendor GmbH, Heidelberg, Germany) were performed in technical duplicates and according to the manufacturer’s instructions in a laboratory at the University of Iceland. The mean Intra-assay CV was < 10% and mean Inter-assay CV < 15% for all assays.

### Subject grouping based on CSF measures

Each subject was classified independently of clinical diagnosis on the basis of CSF T-tau and Aβ_42_ values. T-tau/Aβ_42_ ratio cut-off of 0.52 was chosen based on results from a large memory clinic cohort study [41], giving a sensitivity of 93% for AD and specificity of 83% for controls. A positive CSF AD profile was defined as T-tau/Aβ_42_ ratio > 0.52. The same ratio was also used as a part of the clinical diagnosis of AD, explaining full concordance with CSF AD profile.

### Neuropsychological tests

All subjects underwent a detailed neuropsychological assessment performed by licensed psychologists. Five cognitive domains, commonly affected by aging and AD, were assessed using seven tests (Table 1). For the evaluation of verbal episodic memory, two tests were used. The first, Rey Auditory Verbal Learning Test (RAVLT), consisted of 15 nouns read aloud by the examiner for five consecutive trials. Each trial was followed by a free-recall test. After a 30-min delay, subjects were required to recall the words without being reread the list [42]. The second test was composed of a story [43], which included 25 ideas verbally presented by the examiner. Right after the story was presented (immediate recall), the subject was asked to repeat what they remembered without being given any clues (free recall). Thirty minutes later, subjects were asked to recall the story again (delayed recall). The Rey–Osterrieth complex figure test (ROCF) was used to assess non-verbal episodic memory [42]. The subject was asked to reproduce a complicated line drawing, first by copying it free-hand, second by drawing from memory (immediate recall), and third by drawing it after a 30-min delay (delay recall). Verbal fluency [44] was evaluated with subjects having to produce as many animal names and words starting with the letters H and S as possible in 60 s. Two subtests were used to evaluate processing speed. Part A of The Trail Making Test (TMT-A) [45] required subjects to connect 25 numbered circles positioned randomly on a piece of paper. The first and the most simple part of the Stroop test—Word reading—was also used for the evaluation of the same cognitive domain [46]. Subjects were shown a list of color names (red, green, yellow, or blue), each printed in black ink, and told to read out loud as rapidly as possible. For evaluation of executive functions, The Digit Symbol Substitution Test (DSST), Trail making Test B (TMT-B), and Stroop 4th/3rd parts were used. DSST [47] is a paper-and-pencil test that requires the participant to match symbols to numbers according to a key located at the top of the page. The subject copied the symbol into spaces below a row of numbers. The number of correct symbols within 120 s, constituted the score. TMT-B includes both numbers (1–13) and letters (A-L), with the subject drawing lines between circles, alternating between numbers and letters (1-A-2-B-3-C, etc.). In Stroop—part 4, subjects had to name the color of words when color and meaning were incongruent. Part 3—naming of squares of given colors—was used to control for speed by calculating the ratio between the two parts.

**Table 1:** List of neuropsychological tests administrated

Cognitive domain	Neuropsychological test	Scores (range)
Verbal episodic memory	RAVLT immediate recall	Free recall—the sum of the number of words recalled from trials 1 through 5 (0 to 75)
	RAVLT delayed recall	Delayed free recall—number of words recalled after 30-min delay (0 to 15)
	RAVLT recognition—false positives	Recognition—number of words recognized from a list of 45 words. Number of false positives subtracted from the score (− 30 to 15)
	Story immediate recall	Recall of a story containing 25 ideas (0 to 25)
	Story delayed recall	Recall of a story containing 25 ideas again after 30-min delay (0 to 25)
Non-verbal episodic memory	ROCF immediate recall	Complicated drawing reproduced (0 to 36)
	ROCF delayed recall	Complicated drawing reproduced again after 30-min delay (0 to 36)
Language	Verbal fluency animals	Number of animal names produced in 60 s
	Verbal fluency H+S	Number of words that begin with H/S in 60 s
Processing speed	TMT-A	Time in seconds to connect a set of 25 numbered dots in sequential order
	Stroop test, part I	Time in seconds to read a set of color words written in black
Executive functions	DSST	Number of symbols correctly produced in 120 s
	TMT-B	Time in seconds to connect 25 targets, alternating between numbers and letters
	Stroop 4th/3rd part	Part 3—time in seconds it takes to name squares of given colors Part 4—time in seconds it takes to name the color of a word

*Abbreviations*: *RAVLT* Rey Auditory Verbal Learning Test, *ROCF* Rey–Osterrieth complex figure, *DSST* Digit symbol substitution test, *TMT* Trail Making Test

### Statistical analysis

Descriptive group comparisons were performed using Mann-Whitney *U* tests and chi-square tests for continuous and categorical variables, respectively. Raw values of CSF measures and selected neuropsychological tests (TMT, Stroop test, DSST) were naturally log-transformed to account for a non-normal distribution. Composite scores for each cognitive domain were calculated by averaging neuropsychological test *z*-scores and subsequently converting those scores into *z*-scores. Before the computation of composite scores, *z*-scores for tests measuring reaction time were reversed (TMT, Stroop test, DSST) for the purpose of test consistency (higher scores always indicating better performance). Receiver operating characteristic (ROC) curves were constructed for the differentiation between CSF AD and non-AD profiles. The discrimination abilities of each CSF marker and cognitive domain were compared using the area under the curve (AUC) method, according to DeLong et al. [48]. The AUC is the probability that a randomly selected pair of subjects from each CSF profile group is correctly classified. Stability selection was employed in combination with least absolute shrinkage and selection operator (LASSO) regression for the purpose of identifying stable predictors in multivariable models [49]. LASSO is a penalized approach to multiple regression and especially useful when dealing with multicollinearity (highly correlated predictors). A penalty is introduced, reducing large variance due to multicollinearity in exchange for a tolerable amount of bias. It also performs variable selection as it imposes coefficients of some variables to shrink towards zero. Stable selection is based on resampling the data for avoidance of overfitting, which can be advantageous when dealing with smaller data sets. Instead of fitting one model on a whole sample, many models are fitted on subsamples drawn from it. Stability selection was performed by the use of the function stabsel in the package stabs, implementing the package glmnet for LASSO model fitting [50, 51]. Cut-off value for stable selection was set to 75% (the percentage of times a variable was selected into a model) and per-family error rate (PFER) to 1 for all analyses. Each subsample was half the size of the original one, with 100 subsamples being drawn. LASSO logistic regression was applied for the selection of novel CSF markers and composite tests, most accurately distinguishing between the two CSF profiles. LASSO linear regression was used to select variables, out of CSF markers and demographic variables, predicting with most accuracy the composite *z*-score for each cognitive domain. Two LASSO regressions with a stability selection were performed for each cognitive domain, one which included all subjects and the other, which only included those with a CSF AD profile. Scatter plots were used for visualization of the selected relationships between CSF markers and cognitive domains. Cognitive domain measures were adjusted for age and education before the calculations of corresponding Pearson’s correlations coefficients. For the adjustment, linear regression models were created with each composite test *z*-score as the dependent variable and age and education as independent variables. The residual for each subject was subsequently calculated (observed minus predicted score). Significance values were not adjusted for multiple comparisons, as this study was viewed as explorative with emphasis on discovering relationships. All statistical analyses were performed using R (version 3.6.1, The R Foundation for Statistical Computing).

## Results

### Sample characteristics

Table 2 shows the demographic, pathophysiological, and clinical characteristics of the cohort by CSF profile. There were no significant differences between the groups in age, length of education, novel CSF protein levels, or gender frequencies. Boxplots comparing distributions in CSF protein levels (NFL, YKL-40, S100B, GFAP) between profile groups are presented in Additional file 1, S1a-d. The CSF AD profile group showed significantly worse performance on the MMSE, RAVLT, Story, ROCF immediate recall, and Verbal fluency animal tests compared to the non-AD group (*p* < 0.05).

**Table 2:** Subject demographics, CSF marker levels, and neuropsychological test scores by CSF profile

	CSF profile		
	Non-AD T-tau/Aβ_**42**_ ≤ 0.52 (***n*** = 24)	AD T-tau/Aβ_**42**_ > 0.52 (***n*** = 28)	***p*** value^**a**^
*Demographics*			
Gender (M/F)	16/8	17/11	0.66
Age, years	67 (46–80)	70 (51–84)	0.17
Education, years	14.0 (9–20)	12.5 (6–17)	0.11
*Clinical diagnosis*			
SCI/MCI/AD/LBD	10/13/0/1	2/9/16/1	N/A^b^
*CSF measures*			
Aβ_42_ (pg/ml)	703 (374–2332)	454 (160–822)	N/A^c^
T-tau (pg/ml)	173 (100–722)	416 (132–838)	N/A^c^
NFL (ng/ml)	1.9 (0.9–6.5)	2.5 (1.2–4.5)	0.15
YKL-40 (ng/ml)	165 (83–399)	203 (124–367)	0.12
S100B (pg/ml)	215 (132–335)	230 (129–458)	0.17
GFAP (ng/ml)	1.0 (0.1–7.1)	1.3 (0.5–21.3)	0.09
*Cognitive domains*			
*Global cognition*			
MMSE, score	28 (24–30)	27 (24–30)	0.01
*Verbal episodic memory*			
RAVLT immediate recall, score	36 (23–66)	26.5 (13–51)	0.003
RAVLT delayed recall, score	6.5 (0–15)	1.5 (0–12)	< 0.001
RAVLT recognition-fp, score	9.0 (3–15)	5.5 (−3–15)	0.003
Story immediate recall, score	13.5 (5–17)	8 (1–18)	0.005
Story delayed recall, score	12.0 (1–19)	5.5 (0–16)	0.002
*Non-verbal episodic memory*			
ROCF immediate recall, score	13.3 (0–27)	7.3 (0–26)	0.04
ROCF delayed recall, score	12.8 (0–25)	8.5 (0–26)	0.07
*Language*			
Verbal fluency animal, score	20 (8–33)	14 (4–27)	0.02
Verbal fluency H+S, score	24.0 (14–48)	25.5 (6–63)	1.00
*Processing speed*			
TMT-A, seconds	43.5 (21–133)	48.0 (27–116)	0.22
Stroop—part I, seconds	23.5 (20–42)	24.5 (17–34)	0.64
*Executive functions*			
TMT-B, seconds	109 (44–340)	153 (60–343)	0.06
DSST, score	8.5 (3–51)	7.0 (2–61)	0.24
Stroop 4th/3rd part, seconds	2.1 (1.4–4.0)	2.1 (1.6–5.8)	0.25

*Abbreviations*: *AD* Alzheimer’s disease, *CSF* cerebrospinal fluid, *DDST* Digit Symbol Substitution Test, *fp* false positives, *LBD* Lewy body dementia, *MCI* mild cognitive impairment, *MMSE* Mini-Mental State Examination, *N/A* not applicable, *RAVLT* Rey Auditory-Verbal Learning Test, *ROCF* Rey–Osterrieth complex figure, *SCI* subjective cognitive impairment, *TMT* Trail Making Test

Values are shown as median (range) or as numbers per group, ^a^Mann-Whitney U non-parametric tests used for continuous variables and chi-square tests for categorical variables, *p* values not applicable for ^b^clinical diagnosis due to CSF profiles being part of the diagnostic criteria for AD and ^c^Aβ_42_ and T-tau due to their values used for defining CSF profiles

### Pearson’s correlations between CSF markers

**Fig. 2:**
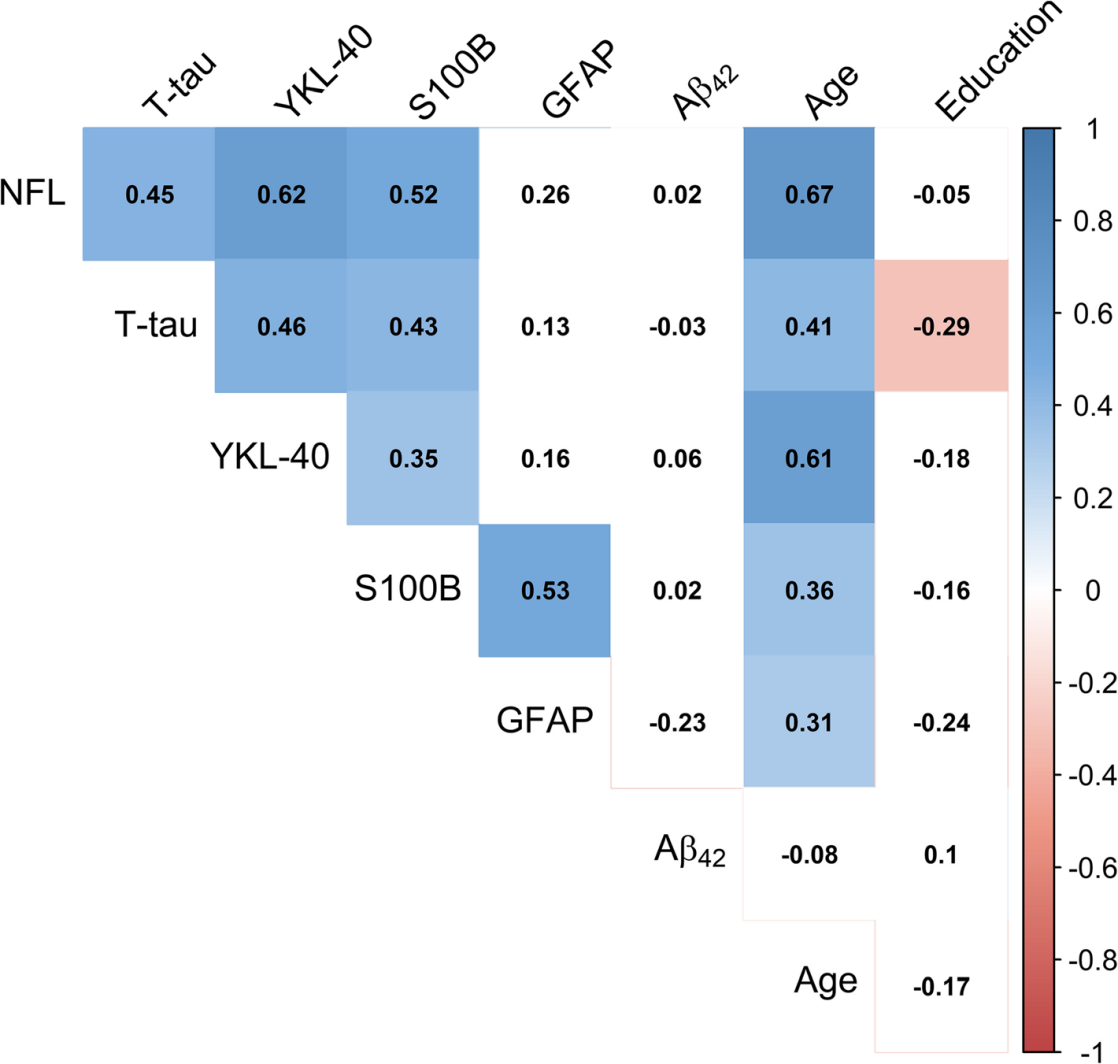
Pearson’s correlation matrix between CSF markers, age, and length of education. Colored squares indicate statistical significance (*p* < 0.05). CSF measures were natural log-transformed

Pearson’s correlations between the CSF markers, age, and length of education are presented in Fig. 2, respectively. Inflammatory markers YKL-40 and S100B and neurodegeneration markers NFL and T-tau all correlated positively and significantly with each other. The highest correlation was found between NFL and YKL-40 (NFL: *r* = 0.62, *p* < 0.001). GFAP did only significantly correlate with the CSF marker S100B (*r* = 0.53, *p* < 0.001). No CSF markers correlated significantly with Aβ_42._ All the CSF markers, except for Aβ_42_, correlated positively with age. Length of education correlated weakly and negatively with T-tau (*r* = − 0.29, *p* = 0.03).

### Accuracy of CSF markers and cognitive domains in distinguishing between CSF profiles

Accuracies for distinguishing between CSF AD and non-AD profiles were based on univariable ROC analyses (Table 3). AUCs for novel CSF markers ranged from 0.61 to 0.64, with a lower limit of each confidence interval below the value of 0.5. In comparison, neuropsychological tests reflecting verbal episodic memory had the highest accuracy compared to other measurements, with all AUCs over 0.70, which is considered fair [52]. The scores for the verbal episodic memory composite test (AUC = 0.80, CI 0.69–0.92) and RAVLT delayed recall (AUC = 0.80, CI 0.68–0.93) distinguished the best between the CSF profile groups. A similar trend in results was found when ROC analyses were stratified by gender (Table S1, Additional file 1), although AUC coefficients were overall higher for women (*n* = 19) compared to men (*n* = 33). LASSO logistic regression with stability selection was performed for the selection of variables distinguishing between the CSF profile groups with the highest consistency. Nine possible predictors could be selected, the four novel CSF markers and the five composite tests presenting each cognitive domain. Only the test reflecting verbal episodic memory was selected as a predictor, with selection frequency (96%) above the cut-off value. All other possible predictors had a much lower selection frequency (≤ 20%).

**Table 3:** Accuracy in distinguishing between CSF AD and non-AD profiles

	Univariable ROC analyses		Multivariable LASSO logistic regression^**b**^
	AUC	95% CI (AUC)*	Stability selection (%)
*CSF measures*^*a*^			
GFAP (ng/ml)	0.64	0.48–0.79	10
YKL-40 (ng/ml)	0.63	0.47–0.78	18
NFL (ng/ml)	0.62	0.45–0.78	2
S100B (pg/ml)	0.61	0.46–0.77	20
*Cognitive domains*			
*Verbal episodic memory*			
Composite *z*-score	0.80	0.69–0.92	96^c^
RAVLT delayed recall, score	0.80	0.68–0.93	–
Story delayed recall, score	0.75	0.62–0.89	–
RAVLT immediate recall, score	0.74	0.61–0.88	–
RAVLT recognition-fp, score	0.74	0.61–0.87	–
Story immediate recall, score	0.73	0.59–0.86	–
*Non-verbal episodic memory*			
Composite *z*-score	0.65	0.50–0.81	14
ROCF immediate recall, score	0.66	0.51–0.81	–
ROCF delayed recall, score	0.65	0.49–0.80	–
*Executive functions*			
Composite *z*-score	0.64	0.49–0.80	16
TMT-B, seconds^a^	0.66	0.50–0.81	–
DSST, score^a^	0.60	0.44–0.75	–
Stroop 4th/3rd part, seconds^*a*^	0.59	0.43–0.75	–
*Language*			
Composite *z*-score	0.60	0.44–0.76	4
Verbal fluency animals, score	0.68	0.54–0.83	–
Verbal fluency H+S, score	0.50	0.34–0.66	–
*Processing speed*			
Composite *z*-score	0.56	0.39–0.72	9
TMT-A, seconds^*a*^	0.60	0.44–0.76	–
Stroop test—part I, seconds^*a*^	0.54	0.38–0.70	–

AUC is the probability that a randomly selected pair of subjects from each CSF profile group is correctly classified

*Abbreviations: AD* Alzheimer’s disease, *AUC* area under curve, *CI* confidence intervals, *CSF* cerebrospinal fluid, *DDST* Digit Symbol Substitution Test, *fp* false positives, *LASSO* Least absolute shrinkage and selection operator*, RAVLT* Rey Auditory-Verbal Learning Test*, ROCF* Rey–Osterrieth complex figure, *TMT* Trail Making Test

*Confidence intervals calculated with DeLong method

^a^Values are natural log-transformed

^b^LASSO logistic regression model was fitted on 100 subsamples, with different predictors (CSF measures and composite test scores) possibly selected into each model. Numbers present the frequency (%) of each possible predictor selected. The per-family error rate (PFER) was set at 1, and the cut-off value at 75% for stability selection

^c^The composite test for verbal episodic memory was the only measure to have selection frequency above the cut-off value

**Fig. 3:**
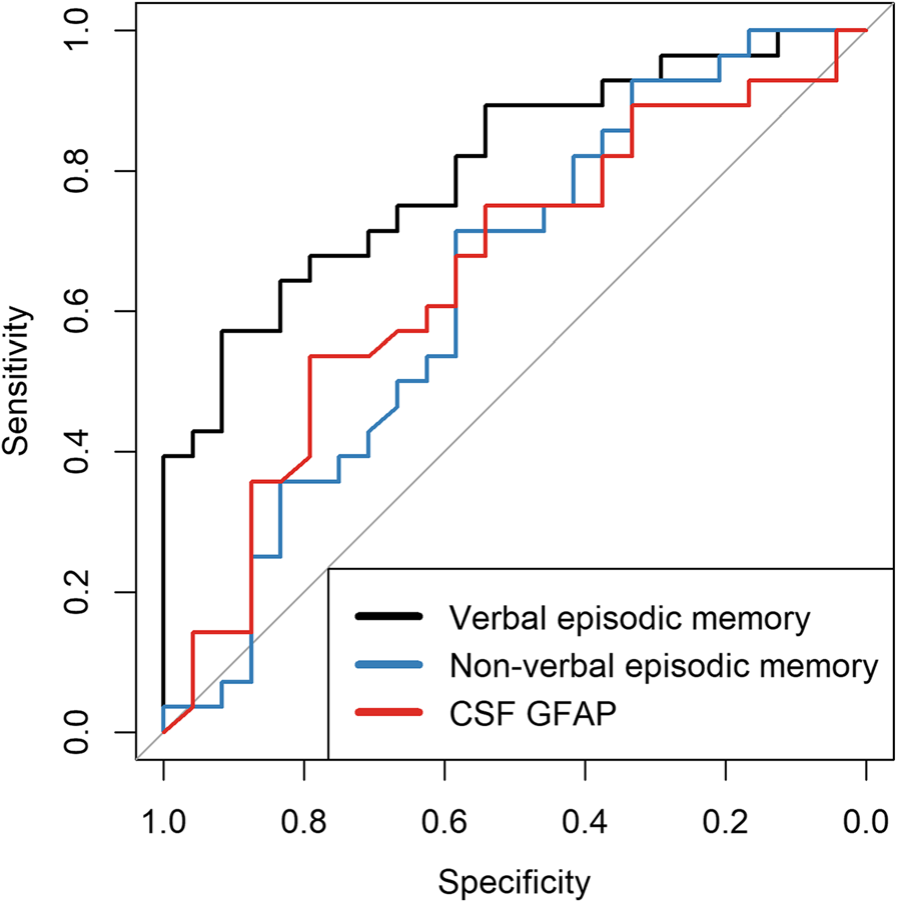
Comparison between ROC curves of the two cognitive domains and the CSF marker with the highest area under the curve (AUC) coefficients

Figure 3 illustrates the ROC curves for the two cognitive domains and the CSF measure with the highest AUC from Table 3. Verbal episodic memory (AUC = 0.80) was superior in distinguishing between CSF AD vs. non-AD profiles compared to non-verbal episodic memory (AUC = 0.65) and CSF GFAP (0.64).

### Selection of predictors for scores on each cognitive domain

**Fig. 4:**
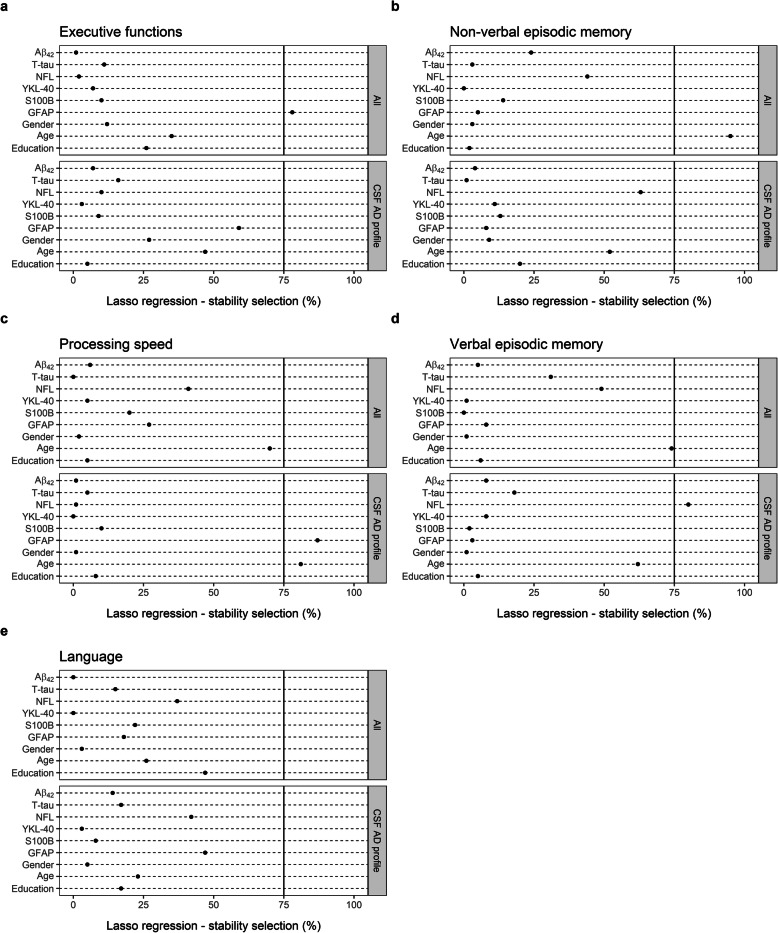
LASSO linear regression—stability selection analyses for prediction of composite *z*-scores reflecting **a** executive functions, **b** non-verbal episodic memory, **c** processing speed, **d** verbal episodic memory, and **e** language. Two analyses were created for each domain, one including all participants (*n* = 52) and the other only the CSF AD profile group (*n* = 28). The cut-off selection value was set at 75% and the per-family error rate (PFER) at 1 for all analyses

LASSO linear regression with a stability selection was applied for identifying a set of variables (CSF markers and demographic variables) predicting cognitive scores with the highest consistency (Fig. 4). Two analyses were performed for each of the five domains, one including all subjects (*n* = 52) and the other only among those with a CSF AD profile (*n* = 28). Variables with stability selection above 75% were considered reliable predictors. GFAP (78%) was selected as a predictor for executive functions (Fig. 4a) and age (95%) as a predictor for non-verbal memory (Fig. 4b) within the whole cohort. Among subjects with a CSF AD profile, GFAP (87%) and age (81%) were selected as predictors for processing speed (Fig. 4c) and NFL (80%) for verbal episodic memory (Fig. 4d). No variables reached the stability selection criteria as predictors of score reflecting language (Fig. 4e).

### Pearson’s correlations between selected CSF markers and cognitive domains

Relationships between CSF measures and cognitive domains, as selected with LASSO regression—stability selection analyses (Fig. 4), were visualized using scatter plots. It is well established that normal aging and level and quality of education can influence cognitive test performance [53]. Composite *z*-scores were therefore adjusted for age and education prior to Pearson’s correlations calculations.

**Fig. 5:**
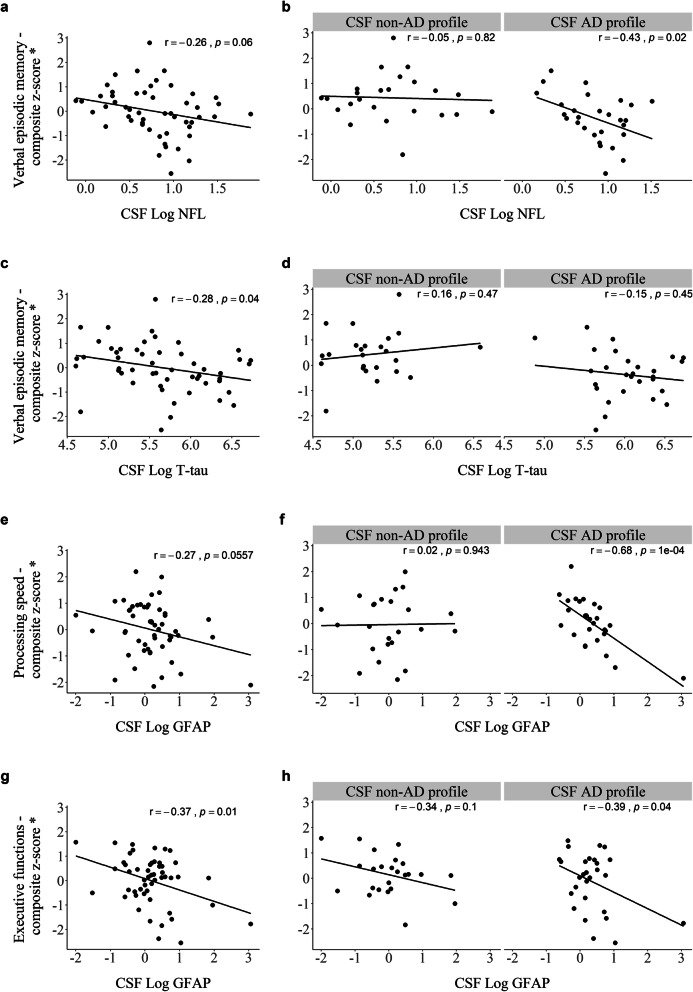
Scatter plots presenting Pearson’s correlations between CSF levels of NFL and verbal episodic memory (**a**, **b**), T-tau and verbal episodic memory (**c**, **d**), GFAP and processing speed (**e**, **f**), and GFAP and executive functions (**g**, **h**) within the whole cohort and by CSF profile. *Cognitive domains were adjusted for covariates (age and education). Without the bottom corner GFAP outlier in the CSF AD profile group, Pearson’s correlations were slightly lower for **f** processing speed (*r* = − 0.58, *p* = 0.001) and **h** executive functions (*r* = − 0.28, *p* = 0.15)

CSF NFL levels did not significantly correlate with verbal episodic memory among all subjects (*r* = − 0.26, *p* = 0.06, Fig. 5a). Analysis by CSF profile (Fig. 5b) revealed moderate, significant correlation among subjects with a CSF AD profile (*r* = − 0.43, *p* = 0.02) compared to none among those without (*r* = − 0.05, *p* = 0.82). Correlations between the NFL levels and individual neuropsychological tests reflecting verbal episodic memory are presented in Additional file 1, S2a-e. T-tau did not reach the selection criteria for any cognitive domain. It is, nonetheless, of interest to compare the results of T-tau to NFL as both proteins are markers of neurodegeneration. The association between T-tau and verbal episodic memory was similar to NFL within the whole cohort (*r* = − 0.28, *p* < 0.04, Fig. 5c) but did not reach significance within the CSF AD group (*r* = − 0.15, *p* = 0.45) when analyzed by CSF profile (Fig. 5d).

Correlation between CSF GFAP levels and processing speed did not reach significance within the whole cohort (*r* = − 0.27, *p* = 0.06, Fig. 5e) or among those with a CSF non-AD profile (*r* = 0.02, *p* = 0.94, Fig. 5f). A moderately strong correlation was, on the other hand, detected among those with a CSF AD profile (*r* = − 0.68, *p* < 0.001, Fig. 5f). A weak, negative correlation was found between CSF GFAP levels and executive functions, both within the whole cohort (*r* = − 0.37, *p* = 0.01, Fig. 5g) and among subjects with a CSF AD profile (*r* = − 0.39, *p* = 0.04, Fig. 5h). The corresponding correlations between CSF GFAP levels with individual neuropsychological tests reflecting processing speed and executive functions are presented in Additional file 1, Fig. S3a-e. Additional file 1 also includes scatter plots identical to those shown in Fig. 5 without adjustment for age and education (Fig. S4a-h) and Pearson’s correlations between CSF markers, age, and education and composite scores of each cognitive domain, both unadjusted and adjusted for age and education (Table S2).

## Discussion

We compared different CSF biomarkers reflecting neurodegeneration (NFL) and inflammation (YKL-40, S100B and GFAP) in relation to core CSF AD markers and cognitive functions in a cohort of subjects at the pre- and early symptomatic dementia stages. While our results indicated that these CSF markers did not accurately distinguish between AD and non-AD CSF profiles, they exhibited different patterns of association with certain cognitive domains, as evaluated by various neuropsychological tests. This pattern was mainly observed among subjects with a CSF AD profile. Within that group, levels of the neurodegeneration marker NFL associated with verbal episodic memory while inflammatory marker GFAP associated with processing speed. In addition, GFAP associated weakly with executive functions within the whole cohort. Overall, these results indicate that CSF NFL and GFAP levels do relate to cognitive functions, specifically among those with a CSF AD profile.

Both CSF NFL and YKL-40 levels correlated with T-tau but not with Aβ_42_, in accordance with previous studies [54–56]; thereby, NFL and YKL-40 levels most likely reflect processes that are independent of Aβ pathology [55, 57, 58]. The putative inflammatory marker, S100B, did show a similar trend as YKL-40 within the whole cohort, correlating strongly with CSF neurodegeneration markers (NFL and T-tau) but not with Aβ_42_ levels. In contrast, GFAP did not correlate with the CSF neurodegeneration markers nor with CSF Aβ_42_ levels. Neither CSF S100B nor GFAP have been much studied in terms of correlation with CSF core AD markers. Hov et al. [32] found similar results among elective surgery patients free from dementia and delirium, with S100B positively correlating with P-tau but not with Aβ_42_ in CSF. Ishiki et al. [33] did not find an association between GFAP and the core AD markers within a sample of healthy subjects and dementia patients. Here we found that CSF NFL, YKL-40, S100B, and GFAP all performed poorly in differentiating between the CSF AD and non-AD profiles. In summary, these results are in accordance with previous findings that have suggested markers NFL, YKL-40, S100B, and GFAP to be not AD specific.

The neuropsychological tests reflecting verbal episodic memory did show the best accuracy in differentiating between the CSF profiles out of all the evaluated cognitive measures and the novel CSF markers. The accuracy was good for the composite score of verbal episodic memory and RAVLT delayed recall test (80%), but fair for all the other verbal episodic memory tests (between 70 and 80%). A recent meta-analysis [59] based on 47 studies has shown that immediate and delayed memory tests consistently show good accuracy (above 80%) for differentiating between AD and healthy controls, especially those involving list recall. Importantly, these studies are based on the clinical diagnosis of AD, while our focus was on the signature of the CSF AD biomarker profile.

CSF markers related in different ways to cognitive measures. Both CSF NFL [56, 60] and YKL-40 [58] have been previously reported to associate with cognitive decline, with correlation found between CSF levels and global cognition assessed by MMSE test scores among AD patients. In the same studies, the correlation did not hold for patients with MCI. Thus, NFL and YKL-40 might not be sensitive to very early changes in cognition in the earliest symptomatic stages of dementia (SCI, MCI) as in more advanced stages. In this study, the relationship between NFL and YKL-40 with different cognitive domains within the whole cohort could not be confirmed. A possible explanation could be that a majority of subjects (*n* = 34) were at the SCI or MCI stages, with 23 of those without a CSF AD profile.

Knowledge regarding the relationship between core CSF biomarkers and cognition remains incomplete. Overall, Aβ_42_ and T-tau appear to associate with memory and executive functions in some studies [61, 62], although results have not been consistent in terms of which cognitive domains they are associated with, which particular tests are most suitable and the strength of relationships in different clinical stages [61, 63, 64]. However, the levels of core CSF marker have shown evidence of reaching a plateau early in the clinical course of the disease and are therefore not considered ideal for tracking the progression of disease at later stages [65].

Increased CSF levels of inflammatory marker GFAP was found weakly associated with worse performance on tests reflecting executive functions, both within the whole cohort and among subjects with CSF AD profile. Few studies have examined the relationship between CSF GFAP levels and cognitive functions. Ishiki et al. [33] did not find an association between CSF GFAP levels and MMSE scores in a sample of healthy subjects and dementia patients. Darreh-Shori et al. [66] also reported no correlation between CSF GFAP levels and MMSE scores among AD patients. As with CSF GFAP, little research has been conducted on the association between CSF S100B levels and cognition. In the same study [66], a weak, positive relationship was found between levels of CSF S100B and MMSE scores within the same patient group.

Associations between selected CSF markers and cognitive domains were also examined within each CSF profile. CSF NFL levels moderately related to verbal episodic memory among those with CSF AD profile but not among those without. Higher levels of CSF GFAP also moderately associated with worse performance on processing speed only within the CSF AD profile group. This is of interest because the CSF markers did not directly relate to the CSF AD profile (ability in discriminating between CSF profiles was poor). This outcome could possibly be explained by the additive effects of distinctive processes on cognitive functions. A previous study [67] showed a similar trend where CSF YKL-40 levels associated with less preservation of global cognition only in individuals with low Aβ levels (Aβ positive). CSF Aβ levels did though not correlate with YKL-40 or cognitive decline, but to brain atrophy in Aβ positive subjects.

This study has several limitations. First, the sample was relatively small, and hence, present findings need to be validated in a larger study. The sample did not include healthy controls, which could underestimate associations between the studied variables. Another limitation of the study is the lack of information about the ApoE genotype. However, it is unlikely that the ApoE genotype affects the outcome as previous studies have suggested that ApoE ε4 status does not influence CSF NFL or YKL-40 levels [19, 68, 69].

## Conclusions

Our findings suggest that levels of CSF markers NFL and GFAP relate to different cognitive profiles at the symptomatic pre- and early dementia stages. The relationships between the levels of NFL with verbal episodic memory and GFAP with processing speed were only observed among those with CSF AD profile, although the CSF markers did not directly relate to the CSF AD profile. These CSF markers could be of potential use as progression markers, monitoring subtle cognitive changes at the earliest symptomatic stages of dementia among those with AD pathology. Further studies with bigger group sizes are needed to validate these results and to evaluate their potential in tracking changes in the more advanced stages of AD and other types of dementia.

## Supplementary information

**Additional file 1: Figure 1.** Levels of CSF NFL, YKL-40, S100B and GFAP by CSF profile. **Table 1.** Univariable ROC analysis for distinguishing between CSF profile groups stratified by gender. **Figure 2.** Pearson’s correlations between levels of CSF NFL with neuropsychological tests reflecting verbal episodic memory by CSF profile. **Figure 3.** Pearson’s correlations between levels of CSF GFAP with neuropsychological tests reflecting processing speed and executive functions by CSF profile. **Figure 4**. Pearson’s correlations between CSF levels of NFL and T-tau with verbal episodic memory and GFAP with processing speed and executive functions, within the whole cohort and by CSF profile. **Table 2.** Pearson’s correlations between CSF markers, age, education and composite z-scores reflecting cognitive domains.

## Data Availability

The data which support this study are not publicly available, but may be provided upon reasonable request.

## Abbreviations

AD: Alzheimer’s disease
AUC: Area under curve
Aβ_42_: Amyloid-β_1–42_
CSF: Cerebrospinal fluid
DSST: Digit Symbol Substitution Test
FP: False positives
GFAP: Glial fibrillary acidic protein
IQCODE: Informant Questionnaire on Cognitive Decline in the Elderly
LASSO: Least absolute shrinkage and selection operator
LBD: Lewy body dementia
MCI: Mild cognitive impairment
MMSE: Mini-Mental State Examination
MTA: Medial temporal lobe atrophy
NFL: Neurofilament light
NFTs: Neurofibrillary tangles
PET: Positron emission tomography
PFER: Per-family error rate
P-tau: Phosphorylated tau
RAVLT: Rey Auditory Verbal Learning Test
ROC: Receiver operating characteristic
ROCF: Rey–Osterrieth Complex Figure
S100B: S100 calcium-binding protein B
SCI: Subjective cognitive impairment
TMT: Trail Making Test
T-tau: Total-tau
