# Supplementary material for: Association of glial and neuronal degeneration markers with Alzheimer’s disease cerebrospinal fluid profile and cognitive functions
Source: Alzheimers Res Ther. 2020 Aug 4;12:92. doi: 10.1186/s13195-020-00657-8 (PMC7404927; doi:10.1186/s13195-020-00657-8)
Supplement: Supplementary file 1 — Additional file 1: Figure 1. Levels of CSF NFL, YKL-40, S100B and GFAP by CSF profile. Table 1. Univariable ROC analysis for distinguishing between CSF profile groups stratified by gender. Figure 2. Pearson’s correlations between levels of CSF NFL with neuropsychological tests reflecting verbal episodic memory by CSF profile. Figure 3. Pearson’s correlations between levels of CSF GFAP with neuropsychological tests reflecting processing speed and executive functions by CSF profile. Figure 4. Pearson’s correlations between CSF levels of NFL and T-tau with verbal episodic memory and GFAP with processing speed and executive functions, within the whole cohort and by CSF profile. Table 2. Pearson’s correlations between CSF markers, age, education and composite z-scores reflecting cognitive domains. [file 13195_2020_657_MOESM1_ESM.docx]

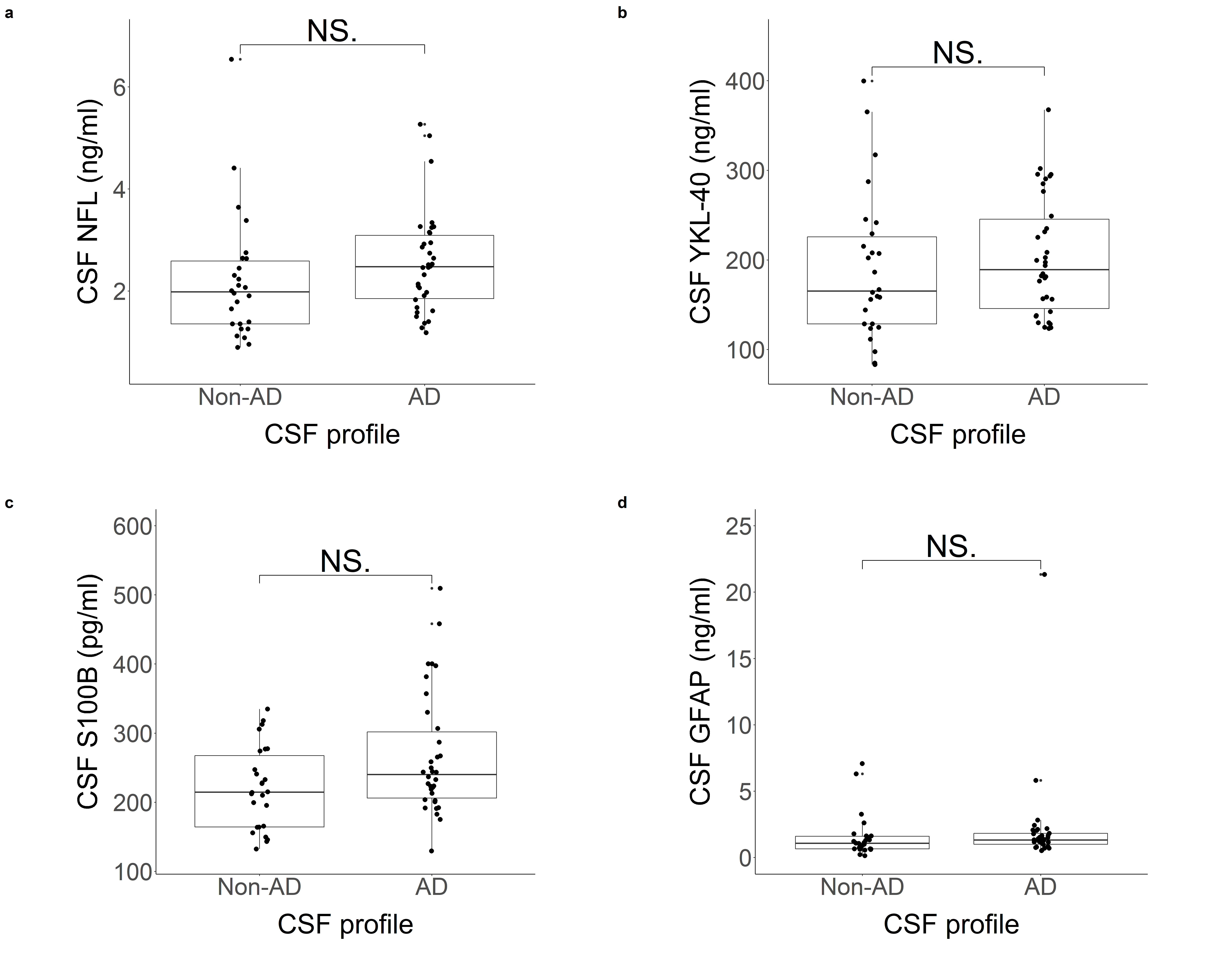


**Fig. S1** Boxplots presenting distributions of CSF measures between CSF profile groups. Levels of CSF a) NFL, b) YKL-40, c) S100B and d) GFAP by CSF profile groups. *NS.* No significance (p>0.05)

**Table S1** Accuracy in distinguishing between AD and non-AD CSF profiles – Univariable ROC analyses stratified by gender

|  |  |  | **Males, n=33** | | **Females, n=19** | |
| --- | --- | --- | --- | --- | --- | --- |
|  |  |  | **AUC** | **95% CI (AUC)*** | **AUC** | **95% CI (AUC)*** |
| *CSF measuresᵃ* | | |  |  |  |  |
|  |  | GFAP (ng/ml) | 0.58 | 0.38-0.79 | 0.76 | 0.54-0.99 |
|  |  | YKL-40 (ng/ml) | 0.48 | 0.28-0.69 | 0.89 | 0.64-1.00 |
|  |  | NFL (ng/ml) | 0.50 | 0.29-0.71 | 0.86 | 0.68-0.98 |
|  |  | S100B (pg/ml) | 0.61 | 0.46-0.77 | 0.72 | 0.46-0.98 |
| *Cognitive domains* | | |  |  |  |  |
|  | *Verbal episodic memory* | |  |  |  |  |
|  |  | Composite z-score | 0.74 | 0.56-0.92 | 0.93 | 0.82-1.00 |
|  |  | RAVLT delayed recall, score | 0.73 | 0.54-0.91 | 0.93 | 0.81-1.00 |
|  |  | Story delayed recall, score | 0.72 | 0.53-0.90 | 0.85 | 0.66-1.00 |
|  |  | RAVLT immediate recall, score | 0.64 | 0.44-0.83 | 0.90 | 0.77-1.00 |
|  |  | RAVLT recognition-fp, score | 0.67 | 0.48-0.86 | 0.86 | 0.69-1.00 |
|  |  | Story immediate recall, score | 0.71 | 0.53-0.89 | 0.78 | 0.57-0.99 |
|  | *Non-verbal episodic memory* | |  |  |  |  |
|  |  | Composite z-score | 0.55 | 0.34-0.75 | 0.83 | 0.61-1.00 |
|  |  | ROCF immediate recall, score | 0.56 | 0.35-0.76 | 0.83 | 0.63-1.00 |
|  |  | ROCF delayed recall, score | 0.55 | 0.34-0.75 | 0.81 | 0.56-1.00 |
|  | *Executive functions* | |  |  |  |  |
|  |  | Composite z-score | 0.56 | 0.36-0.76 | 0.78 | 0.57-1.00 |
|  |  | TMT-B, sec.*ᵃ* | 0.53 | 0.33-0.74 | 0.81 | 0.58-1.00 |
|  |  | DSST, score*ᵃ* | 0.65 | 0.46-0.85 | 0.49 | 0.20-0.78 |
|  |  | Stroop 4^th^/3^rd^ part, sec. *ᵃ* | 0.45 | 0.24-0.65 | 0.72 | 0.48-0.96 |
|  | *Language* | |  |  |  |  |
|  |  | Composite z-score | 0.60 | 0.40-0.80 | 0.58 | 0.29-0.87 |
|  |  | Verbal fluency animals, score | 0.65 | 0.46-0.84 | 0.70 | 0.44-0.97 |
|  |  | Verbal fluency H+S, score | 0.45 | 0.25-0.66 | 0.57 | 0.30-0.85 |
|  | *Processing speed* | |  |  |  |  |
|  |  | Composite z-score | 0.55 | 0.34-0.75 | 0.43 | 0.15-0.71 |
|  |  | TMT-A, sec. *ᵃ* | 0.53 | 0.33-0.74 | 0.72 | 0.48-0.96 |
|  |  | Stroop test – part I, sec. *ᵃ* | 0.57 | 0.36-0.77 | 0.53 | 0.24-0.82 |

*Abbreviations: AD* Alzheimer’s disease, *AUC* Area under curve*, CI* Confidence Intervals, *CSF* Cerebrospinal fluid, *DDST* Digit symbol substitution test, *fp* false positives, *RAVLT Rey Auditory-Verbal Learning Test, ROCF* Rey–Osterrieth complex figure, *SE* Sensitivity*, SP* Specificity, *TMT* Trail Making Test

AUC is the probability that a randomly selected pair of subjects from each CSF profile group is correctly classified, *Confidence intervals calculated with DeLong method*,* ᵅValues are natural log-transformed

**
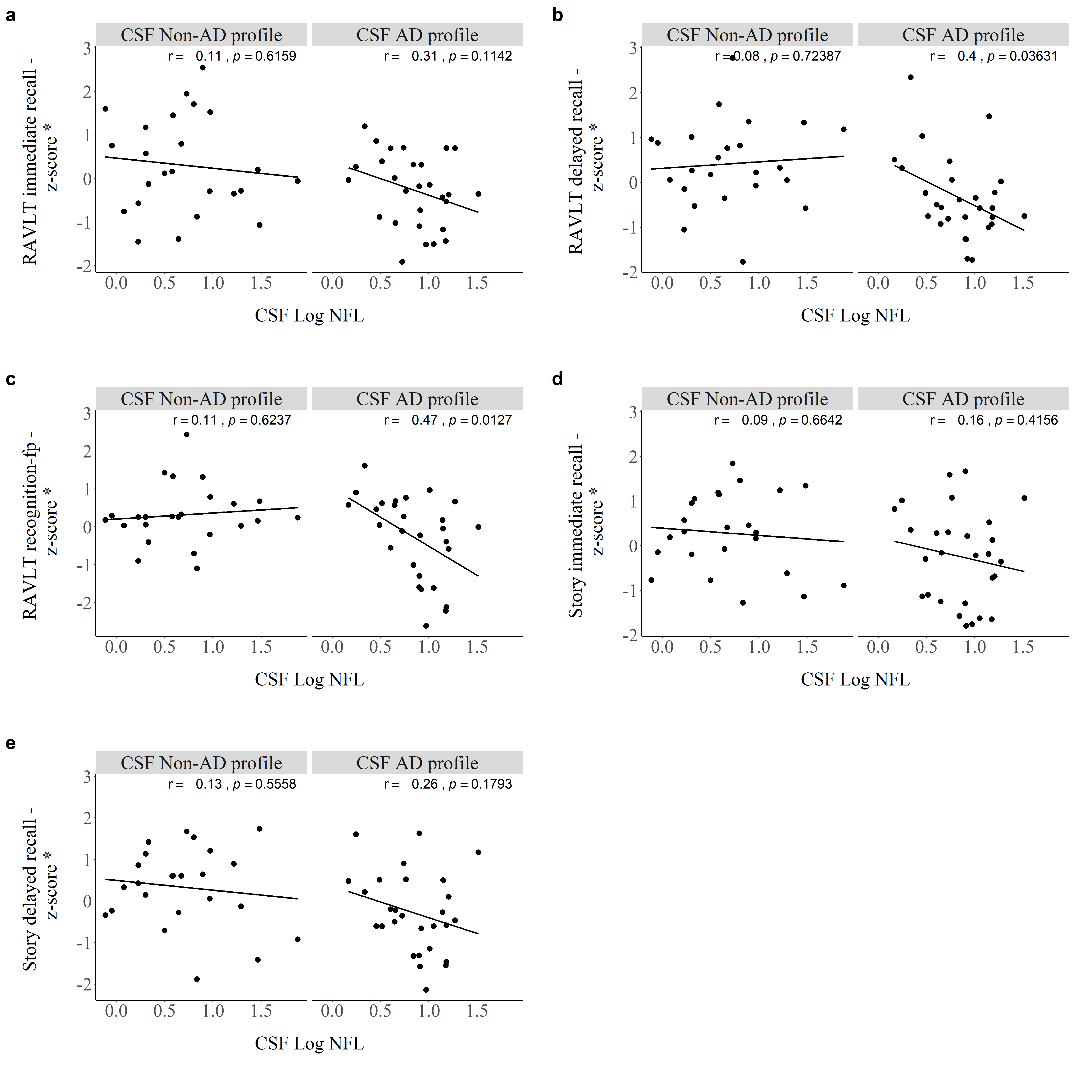
**

**Fig. S2** Pearson‘s correlations between levels of CSF NFL with a) RAVLT immediate recall, b) RAVLT delayed recall, c) RAVLT recognition – fp, d) Story immediate recall and e) Story delayed recall by CSF profile. *Cognitive domains were adjusted for covariates (age and education)


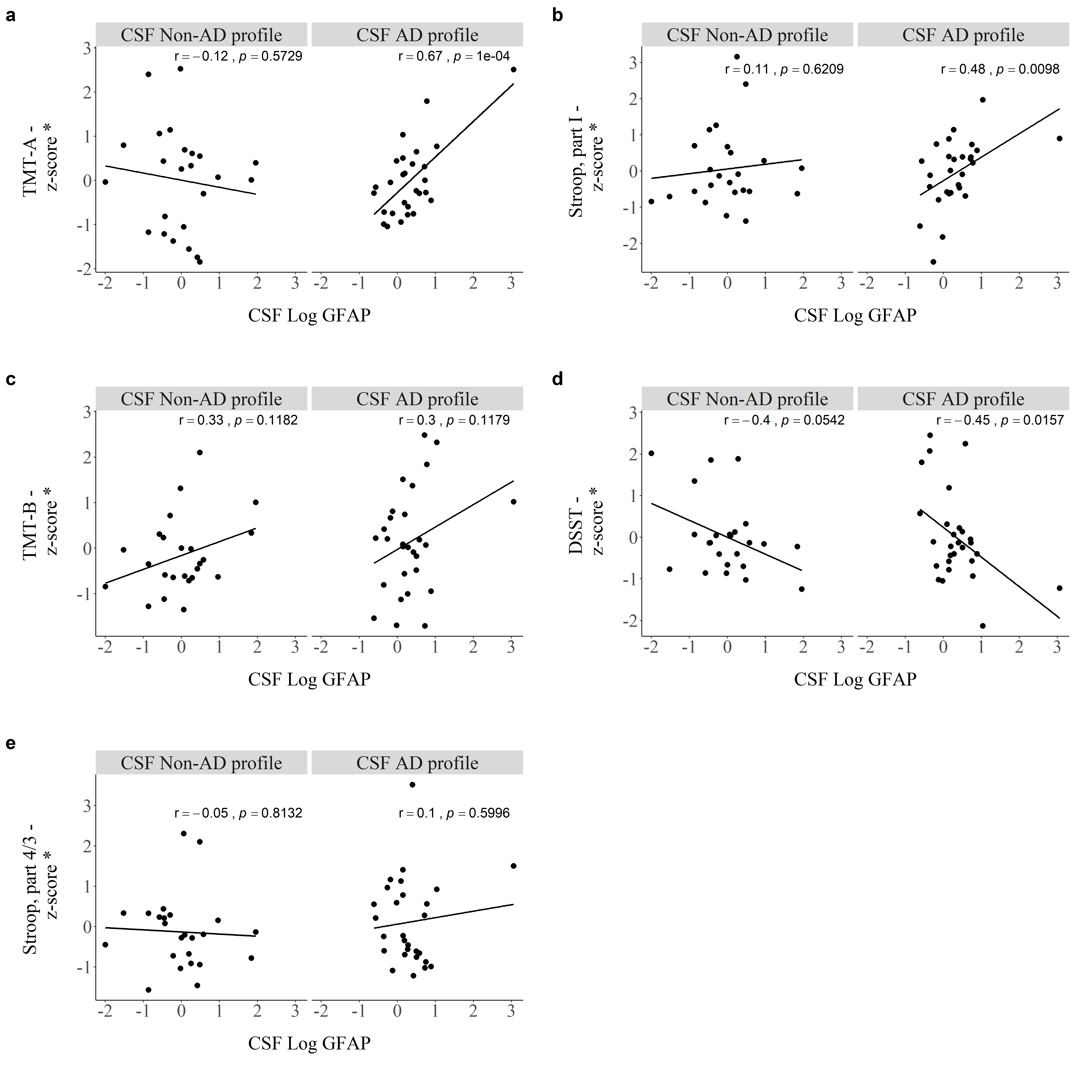


**Fig. S3** Pearson‘s correlations between levels of CSF GFAP with a) TMT-A, b) Stroop, part I,

c) TMT-B, d) DSST and e) Stroop, part 4^th^/3^rd^ by CSF profile. *Cognitive domains were adjusted for covariates (age and education)


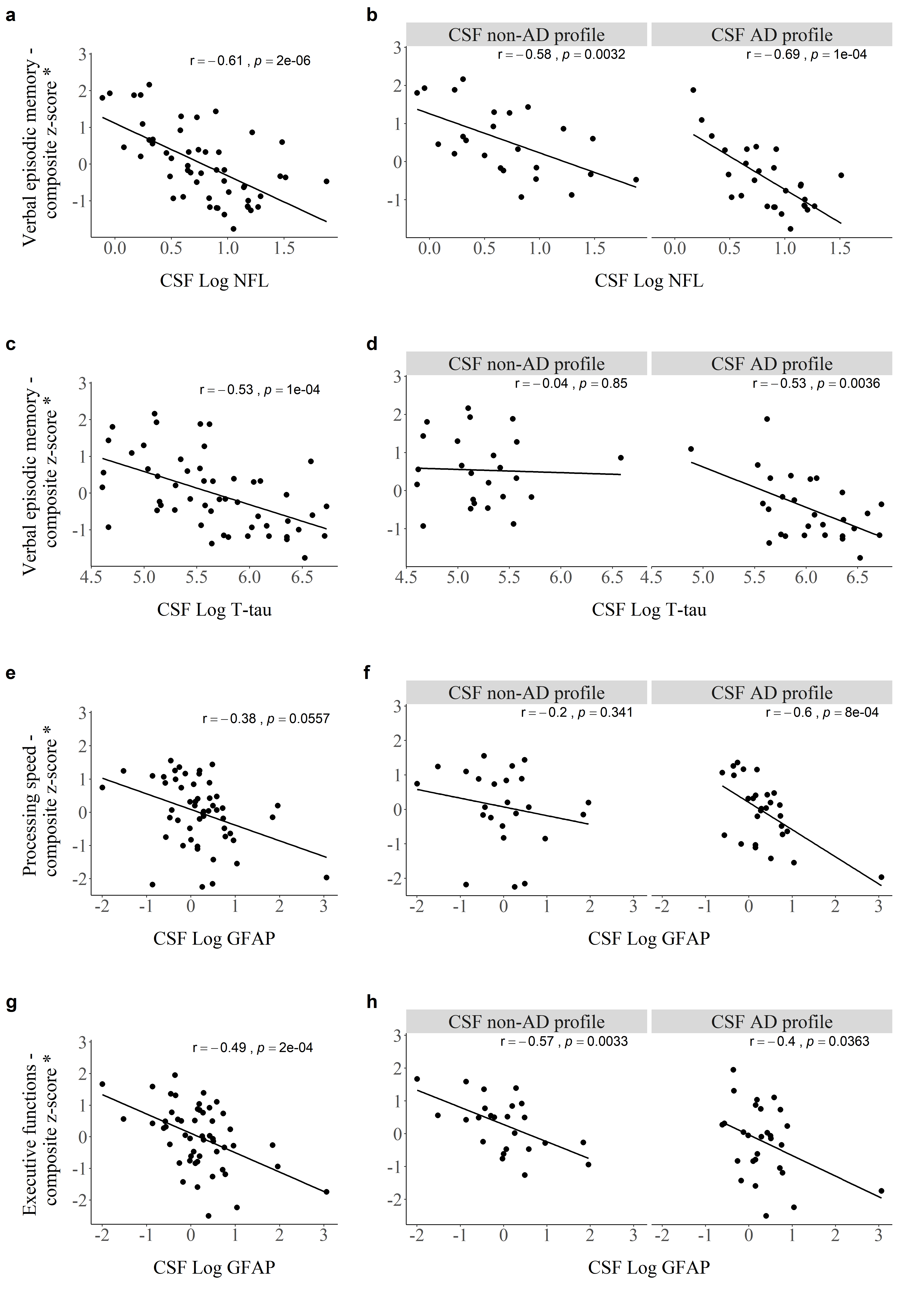


**Fig. S4** Pearson‘s correlations between CSF levels of NFL and verbal episodic memory (a,b), T-tau and verbal episodic memory (c,d), GFAP and processing speed (e,f) and GFAP and executive functions (g,h) within the whole cohort and by CSF profile. *Cognitive domains were not adjusted for covariates (age and education)

**Table S2.** Pearson‘s correlations between CSF markers, age, education and compozite z-scores for each cognitive domain

|  | Verbal episodic memory | | Non-verbal episodic memory | | Language | | Processing  speed | | Executive functions | |
| --- | --- | --- | --- | --- | --- | --- | --- | --- | --- | --- |
|  | Unadj. | Adj. | Unadj. | Adj. | Unadj. | Adj. | Unadj. | Adj. | Unadj. | Adj. |
|  |  |  |  |  |  |  |  |  |  |  |
| Aβ_42_ | 0.23 | 0.22 | 0.26 | 0.26 | -0.04 | -0.11 | 0.15 | 0.14 | 0.12 | 0.08 |
| T-tau | -0.53^***^ | -0.28^*^ | -0.23 | 0.03 | -0.31^*^ | -0.10 | -0.06 | 0.15 | -0.25 | -0.04 |
| NFL | -0.61^***^ | -0.26 | -0.48^***^ | -0.12 | -0.38^**^ | -0.19 | -0.44^**^ | -0.10 | -0.24 | -0.02 |
| YKL-40 | -0.30^*^ | 0.18 | -0.27 | 0.11 | -0.27 | -0.02 | -0.33^*^ | -0.02 | -0.21 | 0.03 |
| S100B | -0.35^*^ | -0.13 | -0.32^*^ | -0.13 | -0.34^*^ | -0.18 | -0.36^**^ | -0.20 | -0.26 | -0.13 |
| GFAP | -0.38^**^ | -0.18 | -0.27 | -0.09 | -0.33^*^ | -0.16 | -0.38^**^ | -0.27 | -0.49^***^ | -0.37** |
| Age | -0.66^***^ | 0.00 | -0.59^***^ | 0.00 | -0.37^**^ | 0.00 | -0.51^***^ | 0.00 | -0.37^**^ | 0.00 |
| Education | 0.39^**^ | 0.00 | 0.16 | 0.00 | 0.40^**^ | 0.00 | 0.03 | 0.00 | 0.33^*^ | 0.00 |

*Unadj.* Unadjusted, *Adj.* Adjusted.

**p*<0.05, ***p*<0.01, ****p*<0.001. Numbers present Pearson‘s correlations coefficients. CSF markers were natural log-transformed. Scores were unadjusted or adjusted for age and education. For the adjustment, linear regression models were created with each test score as the dependent variable and age and education as independent variables. The residual for each subject was subsequently calculated (observed minus predicted score)
